# Impact of pregnant mothers’ previous COVID-19 infection and vaccination on newborns’ serological profiling

**DOI:** 10.3389/fimmu.2025.1526264

**Published:** 2025-09-19

**Authors:** Alaa Masry, Mohammad A. A. Bayoumi, Prem Chandra, Hana J. Abukhadijah, Tawa Olukade, Ismail Abdelhady, Sudheer Thazhe, Ratheesh Paramban, Anoop Sudarsanan, Jeat Abraham, Mohammed Abugubba, Yasser Al-Matar, Maged Soliman Al-Shanwar, Gheyath Khaled Nasrallah, Julin Joseph, Charity Sajor, Sheena Joy, Sheeja George, Nader Al-Dewik, Mai Abdulla Al-Qubaisi

**Affiliations:** ^1^ Neonatal Intensive Care Unit (NICU), Women’s Wellness and Research Center (WWRC), Hamad Medical Corporation (HMC), Doha, Qatar; ^2^ Medical Research Center, Hamad Medical Corporation (HMC), Doha, Qatar; ^3^ Research Department, Women’s Wellness and Research Center (WWRC), Hamad Medical Corporation (HMC), Doha, Qatar; ^4^ Qatar University, Doha, Qatar; ^5^ Obstetrics and Gynecology Department, Women’s Wellness and Research Center (WWRC), Hamad Medical Corporation (HMC), Doha, Qatar

**Keywords:** COVID-19, antibody titer, SARS-CoV-2 coronavirus, vaccination, transplacental immunity, newborn, neonatal intensive care unit, neonate

## Abstract

**Background:**

Pregnant women and newborns are at-risk groups for coronavirus disease 2019 (COVID-19). There is a paucity of evidence to prove the degree of perinatal passive immunity transfer from COVID-19-vaccinated or COVID-19-infected mothers to their newborns.

**Methods:**

We prospectively investigated the vaccination and infection status of 70 women included in the study, as well as the serological characteristics of 72 newborns, to investigate the *in utero* transmission of maternal antibodies against COVID-19 to newborn infants between 2021 and 2022.

**Results:**

A total of 70 pregnant mothers were included in the study after providing signed informed consent. After delivery, cord blood samples were collected from all 72 newborns included in the study. The COVID-19-vaccinated group had significantly higher (p < 0.001) values of both antibodies (NTAb*3.31 and S-RBD*1.15) in the cord blood across both the COVID-19-positive and COVID-19-negative groups. The antibody titres were the lowest in mothers who were not vaccinated and the highest in those who received three vaccination doses (p < 0.001). Multivariate linear regression analysis was performed using dependent variables NTAb*3.31 and S-RBD*1.15 antibodies and independent predictor variables nationality, infant’s gender, COVID-19 vaccination status, and COVID-19 test status; the multivariate linear regression analysis results indicated that vaccination against COVID-19 remained a potential significant (p < 0.0001) predictor for both NTAb*3.31 and S-RBD*1.15 antibodies after adjusting other potential predictor variables.

**Conclusions:**

In our study, we found significantly higher titres of NTAb*3.31 and S-RBD*1.15 antibodies in newborns’ cord blood whose mothers had previous COVID-19 infection or received COVID-19 vaccination; however, these titres were higher in the case of vaccination than previous infection. The more doses of vaccine received, the higher the antibody levels in newborns’ cord blood. This indicates transplacental immunity transmission from mothers to their newborns after previous COVID-19 vaccination or infection.

## Introduction

Coronavirus disease 2019 (COVID-19), caused by severe acute respiratory syndrome coronavirus 2 (SARS-CoV-2), is highly infectious. SARS-CoV-2 was first described by Huang et al. (1), and it continues to pose a global public health concern (2).

Pregnant women and newborns are at-risk groups for COVID-19 (3, 4). The COVID-19 virus was detected in various tissues of the body as well as body fluids, including the umbilical cord, amniotic fluid, and placental tissue and placental circulation. Maternal antibodies have dual effects. They supply passive protection and prime the immune system of the child for subsequent exposure to an antigen. In humans, maternal antibodies wane over 6–12 months. The kinetics of maternal antibody decline are correlated with the amount of maternal antibody present in the neonate after birth. Higher titres persist for a longer time. Furthermore, the number of antibodies could be pathogen- and dose-dependent (5).

During an infection, the immune response is activated to produce antibodies by plasma cells (activated B cells) specific to spike proteins (or other viral structural proteins). Antibodies specific to S1 could neutralise and block the attachment and fusion of SARS-CoV-2 to the host cell before its replication within the lungs and other tissues, including the gastrointestinal tract (6).

Tests for IgG and IgM antibodies for SARS-CoV-2 became available in February 2020. On March 4, 2020, the seventh edition of The New Coronavirus Pneumonia Prevention and Control Protocol for SARS-CoV-2 was released by the National Health Commission of the People’s Republic of China and added serological diagnostic criteria (7, 8).

A recent study found that in a woman positive for SARS-CoV-2, RNA was initially detected in nasopharyngeal samples but not in serum or breast milk samples. The presence of SARS-CoV-2 RNA does not mean vertical transmission to the newborn, as this transmission remains very unlikely (9–11). However, limited studies have been conducted to prove perinatal passive immunity transfer from COVID-19-infected or COVID-19-vaccinated mothers to their newborns. There has been no evidence of mother-to-newborn vertical transmission of SARS-CoV-2 (3, 12), and little is known about susceptibility and immunological characteristics.

In this study, we aimed to prospectively investigate the immunological characteristics of newborns delivered to mothers with previous COVID-19 infection or vaccination for the possibility of acquiring passive transplacental transmission immunity.

## Methods

This prospective observational study was conducted in the Neonatal Intensive Care Unit (NICU) at the Women’s Wellness and Research Center (WWRC), Hamad Medical Corporation (HMC), Doha, Qatar. The WWRC is Qatar’s main hospital for tertiary care obstetrics and neonatal–perinatal services. The NICU in the WWRC is equipped with 128 tertiary-level beds. The WWRC has >18,000 deliveries per year. The NICU has >3,000 admissions per year.

This prospective study was conducted over 2 years in 2021 and 2022 after obtaining institutional review board (IRB) approval (Protocol Number: MRC-01-20-1132) in full conformance with the principles of the Declaration of Helsinki and Good Clinical Practice (GCP) within the laws and regulations of the Ministry of Public Health in Qatar.

We prospectively investigated the vaccination and infection status of the 70 women included in the study, as well as the serological characteristics of the 72 newborns, to investigate the *in utero* transmission of maternal antibodies against COVID-19 to newborn infants.

Informed consent was obtained from pregnant women by a research team member before the participation of the women and their newborns. The consent was obtained while the pregnant mothers were in the hospital before delivery, and enough time was given according to their preference and acceptance to participate in this study. The confidentiality and privacy of all participants were protected, and patient identifiers were not disclosed.

Knowing the maternal COVID-19 infection and vaccination status provided us with data and directed us to further investigate newborn serological profiling. We believed that these data would help in proving or discouraging the notion of passive immunity transmission from pregnant mothers to their newborns after COVID-19 infection or vaccination. Nasopharyngeal aspirate samples were collected from the mothers upon hospital admission for delivery. A cord blood sample from each newborn was evaluated. A 4-mL volume of cord blood was collected after delivery in an ethylenediaminetetraacetic acid (EDTA) tube by the maternity nurse in the labour room/operating theatre of the WWRC. The cord blood samples were tested for neutralising antibodies (NTAbs) and spike-receptor binding domain (S-RBD) antibodies. All collected samples were kept in a refrigerator (for a maximum period of 12 hours) until collected by the research team and sent to the laboratory.

### Laboratory methods

The cord blood was tested by rapid lateral flow immunochromatography assay (LFA; Lionex, Braunschweig, Germany). The level of antibody was measured by serial dilution of the specimen, and then the titration score was measured. All samples positive by ELISA and lateral flow assay were tested for the presence of neutralising antibodies (NTAbs) using the surrogate virus neutralisation assay (GenScript, New Jersey, USA) as described by the manufacturer. The ELISA and the nAb assay were performed in the Qatar University Laboratory. The level of neutralising antibody was measured by calculating the present inhibition score. A sample with more than 20% inhibition score was considered positive for NTAbs. All mothers were tested using the same assays to confirm vertical transmission from mother to infant.

Data collected included gender, nationality, maternal year of birth, date of delivery, collection date of the cord blood, maternal vaccination status against COVID-19, type of vaccine, number of vaccine doses, timing and date of vaccination, date of COVID-19 infection, symptoms of COVID-19 infection, and COVID-19 antibody test results, titres, and interpretation for both the mother and neonate. A comparison and a correlation were performed between the level of maternal antibodies and severity of infection, date of infection, timing during pregnancy, age of the pregnant woman, age of the infant, and stability of antibodies after birth (peak and the waning time). Data were coded and collected in an Excel sheet specifically designed for the study purpose. No patients’ identifiers were disclosed.

### Inclusion criteria

1. We included all pregnant mothers who previously had a COVID-19 infection, were vaccinated, or were not infected or vaccinated in 2021 and 2022.
2. We included all newborns with gestational age >34 weeks delivered to the previously mentioned mothers in 2021 and 2022.

### Exclusion criteria

1. We excluded mothers receiving immunosuppressive medications and those who received Intravenous Immunoglobulins (IVIG) before delivery.
2. We excluded newborns suspected of having an immunodeficiency disease and preterms with gestational age <34 weeks.

### Statistical analysis

Descriptive statistics were used to summarise and determine the participants’ characteristics and data distribution. The normally distributed data were presented as mean with standard deviation (SD), whereas median and inter-quartile range (IQR) were used for skewed or non-normal data distribution. Categorical data were summarised using frequencies and respective percentages. Various parameters measured on categorical scales and their association with categorised NTAb and S-RBD antibody levels in cord blood (non-reactive and reactive) and the number of vaccination doses were evaluated using the chi-square (χ^2^) test, Fisher’s exact, or Yates’s corrected chi-square tests, as appropriate. Quantitative data outcomes (NTAb and S-RBD antibody levels in cord blood) between the two independent groups (reactive and non-reactive) were compared using a non-parametric Mann–Whitney U test.

Furthermore, linear regression analysis was performed using dependent variables NTAb*3.31 and S-RBD*1.15 antibody levels in cord blood and independent predictor variables nationality, infant’s gender, vaccination status against COVID-19, and COVID-19 test status; the results were reported using respective values of regression coefficients along with 95% confidence interval (CI). Receiver operating characteristic (ROC) curves were calculated to derive the best suitable cutoff values for both antibodies NTAb*3.31 and S-RBD*1.15 against vaccination status; sensitivity, specificity, and positive and negative likelihood ratio values were computed to evaluate and assess predictive accuracy. Because sensitivity and specificity were considered equally important, the best cutoff points were determined using Youden’s index, which maximises sensitivity and specificity. ROC curves provide a comprehensive and visually attractive way to summarise the accuracy of predictions. The ROC curve shows the tradeoff between sensitivity and specificity. It is a widely adopted method to detect the performance of a developed test, which classifies subjects into two categories: those who received and those who did not receive the COVID-19 vaccination. A box plot was constructed to depict the distribution of both antibodies (NTAb*3.31 and S-RBD*1.15) in the cord blood across the status of COVID-19 and vaccination groups. A two-sided p-value <0.05 was considered statistically significant. All statistical analyses were conducted using statistical software packages SPSS version 29.0 (IBM Corp., Armonk, NY, USA) and Epi Info (Centers for Disease Control and Prevention, Atlanta, GA, USA).

## Results

The mothers were classified based on their COVID-19 infection and vaccination status, and newborns were investigated for possible passive immunity transmission through the placenta.

A total of 70 pregnant mothers were included in the study after providing signed informed consent. After delivery, cord blood samples were collected from all 72 newborns included in the study. The samples were sent to the laboratory and investigated for specific COVID-19 antibodies. **Table 1** shows the descriptive statistics of the study population.

**Table 1:** Descriptive statistics of the study population.

Parameter	Number and Percentage
Nationality	
Qatari	20 (27.8%)
Non-Qatari	52 (72.2%)
Baby’s gender	
Male	32 (45.1%)
Female	39 (54.9%)
Have you ever been vaccinated against COVID-19?	
Non-vaccinated	28 (38.9%)
Vaccinated	44 (61.1%)
If yes, which COVID-19 vaccine did you take?	
Pfizer	32 (72.7%)
Moderna	12 (27.3%)
How many doses did you take?	
1	2 (4.5%)
2	38 (86.4%)
3	4 (9.1%)
Have you ever tested positive for COVID-19?	
COVID-19 negative	46 (63.9%)
COVID-19 positive	26 (36.1%)
Symptomatic/asymptomatic	
Symptomatic	20 (80.0%)
Asymptomatic	5 (20.0%)
Has the infant tested positive for COVID-19?	
Negative	13/13 (100.0%)
NTAb*3.31 antibody levels in the cord blood	Mean 993.9 (SD 1,079.7) Median 935.3 (IQR 35–1,548.4)
NTAb antibody level categorisation in the cord blood	
Non-reactive	19 (26.4%)
Reactive	53 (73.6%)
S-RBD*1.15 antibody levels in the cord blood	Mean 909.8 (SD 1,053.8) Median 684.7 (IQR 41.7–1,325.7)
S-RBD antibody level categorisation in the cord blood	
Non-reactive	15 (20.8%)
Reactive	57 (79.2%)

IQR, inter-quartile range.

Some values were observed to be missing for some parameters, and hence, the total may not equal n = 72 for such parameters; the respective % values were computed using non-missing observations.

**Figure 1:**
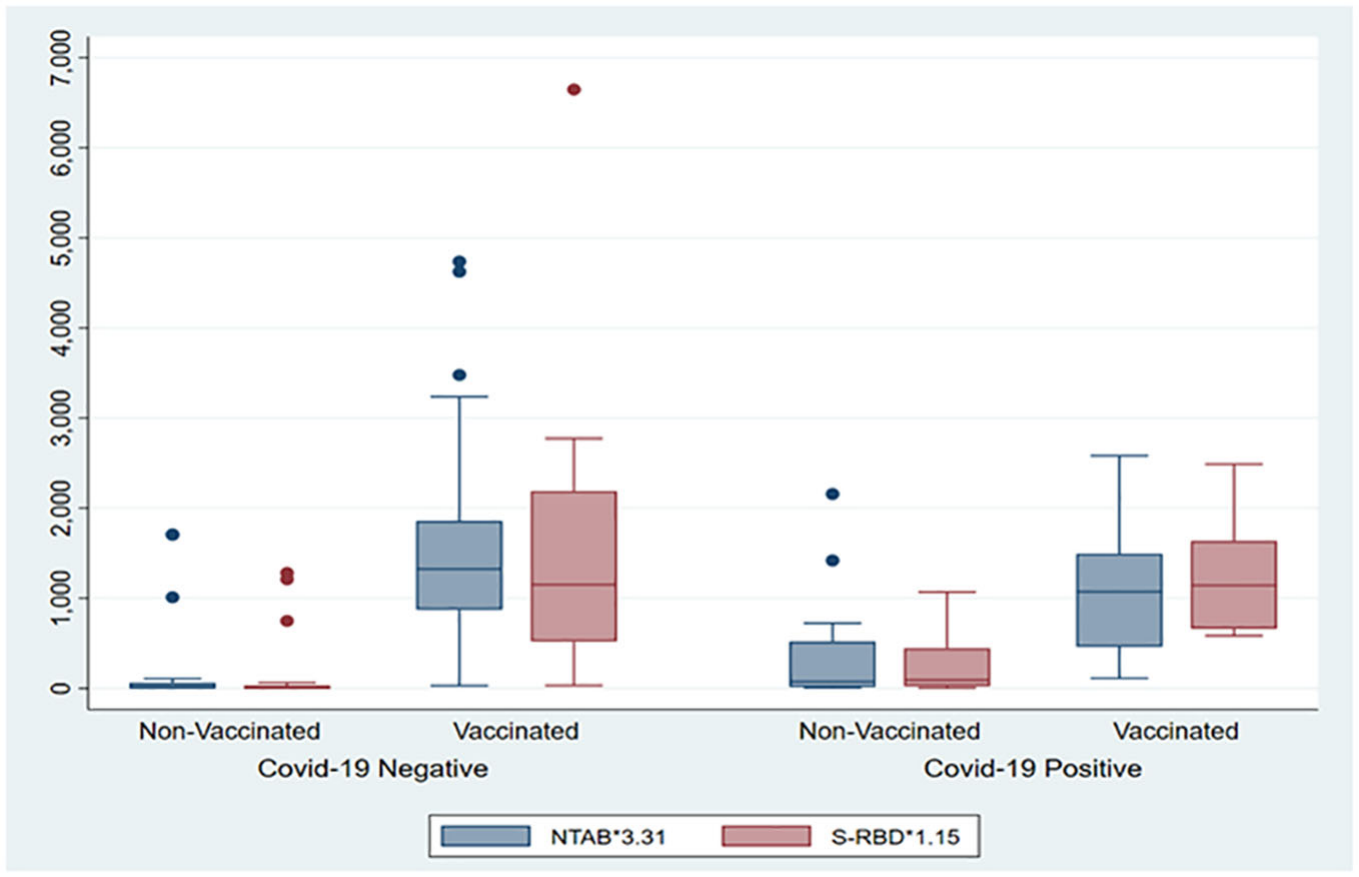
The box plot depicts the distribution of both antibodies (NTAb*3.31 and S-RBD*1.15) in the cord blood across the status of COVID-19 and vaccination groups.

The box plot in **Figure 1** shows that the COVID-19-vaccinated group had significantly higher (p < 0.001) values of both antibodies (NTAb*3.31 and S-RBD*1.15) in the cord blood across both the COVID-19-positive and COVID-19-negative groups. **Table 2** shows NTAb*3.31 and SRBD* 1.15 antibody levels in the cord blood of the study population.

**Table 2:** NTAb*3.31 and S-RBD*1.15 antibody levels in the cord blood of the study population.

Parameters	Total (n = 72)	NTAb antibody levels			S-RBD antibody levels		
		Non-reactive (n = 19)	Reactive (n = 53)	p-Value	Non-reactive (n = 15)	Reactive (n = 57)	p-Value
Nationality				0.446			0.914
Qatari	20 (27.8)	4 (21%)	16 (30%)		4 (27%)	16 (28%)	
Non-Qatari	52 (72.2%)	15 (79%)	37 (70%)		11 (73%)	41 (72%)	
Baby’s gender				0.433			0.304
Male	32 (45.1%)	7 (37%)	25 (48%)		5 (33%)	27 (48%)	
Female	39 (54.9%)	12 (63%)	27 (52%)		10 (67%)	29 (52%)	
Have you ever been vaccinated against COVID-19?				<0.001			<0.001
Non-vaccinated	28 (38.9%)	17 (89%)	11 (21%)		15 (100%)	13 (23%)	
vaccinated	44 (61.1%)	2 (11%)	42 (79%)		0 (0%)	44 (77%)	
If yes, which COVID-19 vaccine did you take?				0.998			n.a.
Pfizer	32 (72.7%)	2/2 (100%)	30/42 (71.4%)		0 (0%)	32/44 (72.7%)	
Moderna	12 (27.3%)	0 (0%)	12/42 (28.6%)		0 (0%)	12/44 (27.3%)	
How many doses did you take?				0.848			n.a.
1	2 (4.5%)	0 (0%)	2/42 (4.8%)		0 (0%)	2/44 (4.6%)	
2	38 (86.4%)	2/2 (100%)	36/42 (85.7%)		0 (0%)	38/44 (86.3%)	
3	4 (9.1%)	0 (0%)	4/42 (9.5%)		0 (0%)	4/44 (9.1%)	
Have you ever tested positive for COVID-19?				0.301			0.144
COVID-19 negative	46 (63.9%)	14 (74%)	32 (60%)		12 (80%)	34 (60%)	
COVID-19 positive	26 (36.1%)	5 (26%)	21 (40%)		3 (20%)	23 (40%)	
Symptomatic/asymptomatic				0.997			0.504
Symptomatic	20 (80%)	3/4 (75%)	17/21 (81%)		2/3 (67%)	18/22 (82%)	
Asymptomatic	5 (20%)	1/4 (25%)	4/21 (19%)		1/3 (33%)	4/22 (18%)	
Antibody levels in the cord blood Median (IQR)	935.3^‡^ (35–1,548.4) 909.8^†^ (41–1,325.7)	6.62 (6.6–15.9)	1,271 (435–1,709)	<0.001	3.45 (3.45–3.45)	1,001 (433.3–1,603)	<0.001

^‡^NTAb*3.31 antibody levels.

^†^S-RBD*1.15 antibody levels.

IQR, inter-quartile range.

Some values were observed to be missing for some parameters, and hence, the total may not equal n = 72 for such parameters; the respective % values were computed using non-missing observations.

**Figure 2:**
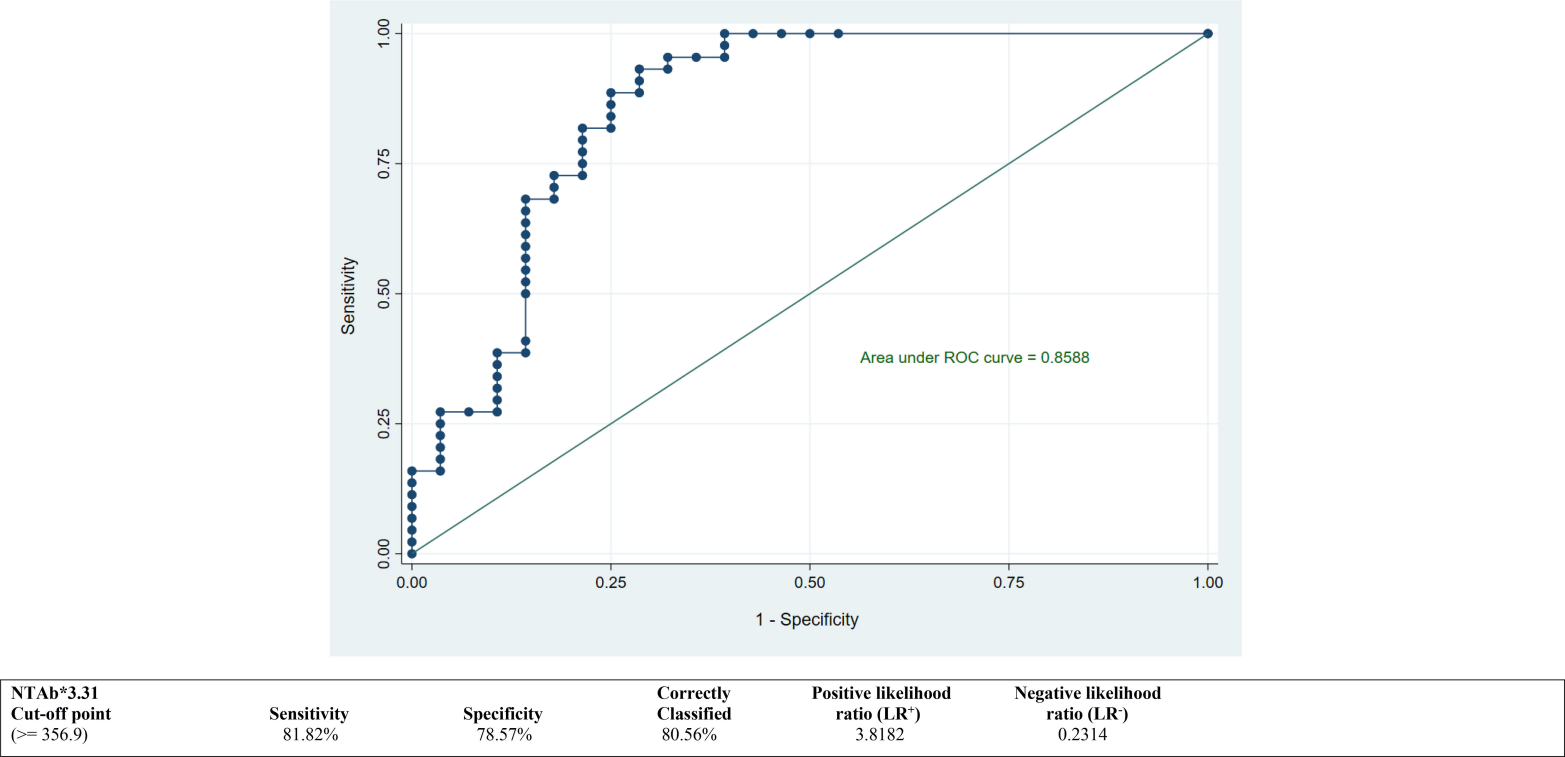
ROC curve to determine an optimum cutoff value for NTAb*3.31 antibodies in the COVID-19-vaccinated group. ROC, receiver operating characteristic.

**Figure 3:**
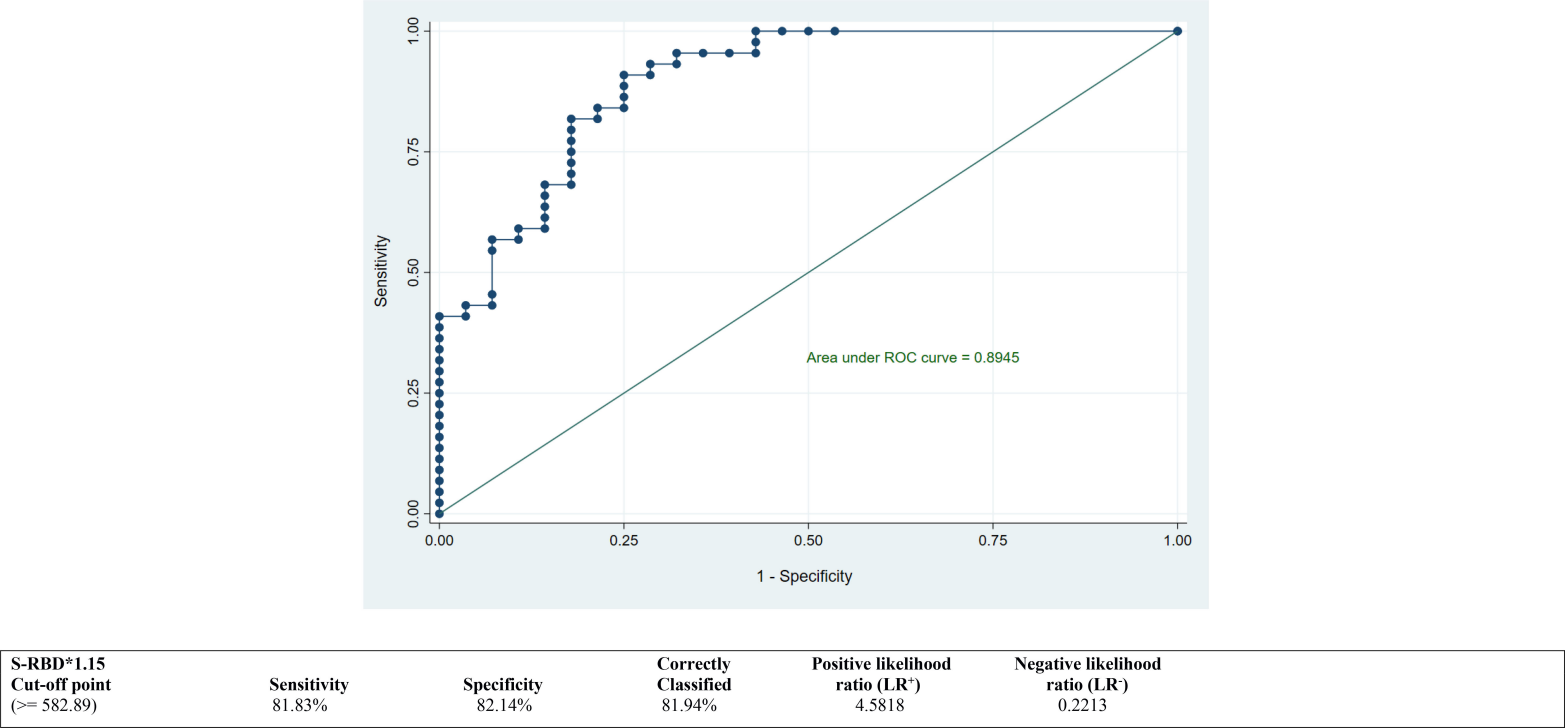
ROC curve to determine an optimum cutoff value for S-RBD*1.15 antibodies in the COVID-19-vaccinated group. ROC, receiver operating characteristic.

ROC curves were calculated to derive the best suitable cutoff values for both antibodies NTAb*3.31 and S-RBD*1.15 and to assess predictive accuracy. ROC curves depicted in **Figures 2** and **3** demonstrate an optimum cutoff value for both antibodies NTAb*3.31 and S-RBD*1.15 in the COVID-19-vaccinated group with their respective Area Under the Curve (AUC) values of 0.85 (95% CI 0.76, 0.95) and 0.89 (95% CI 0.82, 0.97), and high level of various diagnostic indices such as sensitivity, specificity, accuracy, and positive and negative likelihood ratio values indicates that the derived cutoff values for both antibodies exhibit high predictive accuracy.

We found that the mother’s number of vaccination doses is significantlyt and directly proportional to the antibody levels in the cord blood. The antibody titres were the lowest in those who were not vaccinated and the highest in mothers who received three vaccination doses (p < 0.001). The number of vaccination doses in relation to other parameters is shown in **Table 3**.

**Table 3:** Number of vaccination doses in relation to other study parameters.

Parameters	Total N = 72	Not vaccinated N=28	One vaccine dose N=2	Two vaccine doses N=38	Three vaccine doses N=4	p-Value
Nationality						
Qatari	20 (28%)	7 (25%)	0 (0%)	13 (34%)	0 (0%)	0.362
Non-Qatari	52 (72%)	21 (75%)	2 (100%)	25 (66%)	4 (100%)	
Baby’s gender						
Male	32 (45%)	10 (36%)	2 (100%)	18 (49%)	2 (50%)	0.301
Female	39 (55%)	18 (64%)	0 (0%)	19 (51%)	2 (50%)	
Have you ever been vaccinated against COVID-19?						
Non-vaccinated	28 (39%)	28 (100%)	0 (0%)	0 (0%)	0 (0%)	<0.001
Vaccinated	44 (61%)	0 (0%)	2 (100%)	38 (100%)	4 (100%)	
If yes, which COVID-19 vaccine did you take?						
Pfizer	32 (73%)	0 (0%)	2 (100%)	27 (71%)	3 (75%)	<0.001
Moderna	12 (27%)	0 (0%)	0 (0%)	11 (29%)	1 (25%)	
Have you ever tested positive for COVID-19?						
COVID-19 negative	46 (64%)	16 (57%)	1 (50%)	27 (71%)	2 (50%)	0.594
COVID-19 positive	26 (36%)	12 (43%)	1 (50%)	11 (29%)	2 (50%)	
Symptomatic/asymptomatic						
Symptomatic	20 (80%)	8 (67%)	1 (100%)	10 (100%)	1 (50%)	0.157
Asymptomatic	5 (20%)	4 (33%)	0 (0%)	0 (0%)	1 (50%)	
Has the infant tested positive for COVID-19?						
COVID-19 negative	13 (18%)	8 (29%)	1 (50%)	3 (8%)	1 (25%)	0.1
NA	59 (82%)	20 (71%)	1 (50%)	35 (92%)	3 (75%)	
NTAb*3.31 antibody levels in the cord blood*	935.31	15.72	1,352.47	1,324.00	1,142.45	<0.001
	(35.1–1,548.4)	(6.62–270.6)	(252.22–1,352.5)	(447.7–1,711.2)	(712.1–2,745.1)	
NTAb antibody level categorisation in the cord blood?						
Non-reactive	19 (26%)	17 (61%)	0 (0%)	2 (5)	0 (0%)	<0.001
Reactive	53 (74%)	11 (39%)	2 (100%)	36 (95%)	4 (100%)	
S-RBD*1.15 antibody levels in the cord blood*	909.8	16.01	887.45	1,150.00	1,510.25	<0.001
	(41.71–1,325.7)	(3.45–305.7)	(188.02–887.5)	(630.7–1,984.1)	(927.3–1,927.5)	
S-RBD antibody level categorisation in the cord blood?						
Non-reactive	15 (21%)	15 (54%)	0 (0%)	0 (0%)	0 (0%)	<0.001
Reactive	57 (79%)	13 (46%)	2 (100%)	38 (100%)	4 (100%)	

*Median and IQR: Inter-quartile-range.

Some values were observed to be missing for some parameters, and hence, the total may not equal n = 72 for such parameters; the respective % values were computed using non-missing observations.

**Figure 4:**
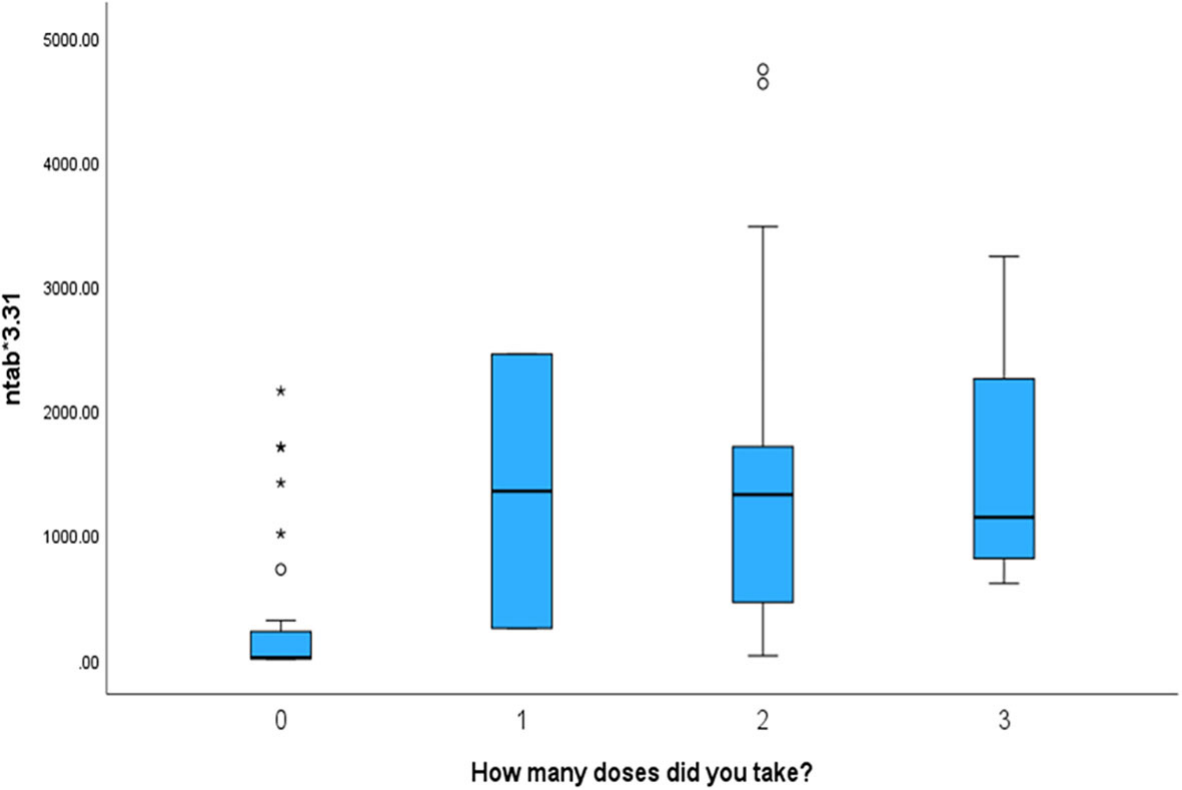
The box plot depicts that mothers who received more vaccination doses against COVID-19 had higher values of NTAb*3.31 antibodies in the serum of their offspring.

**Figure 5:**
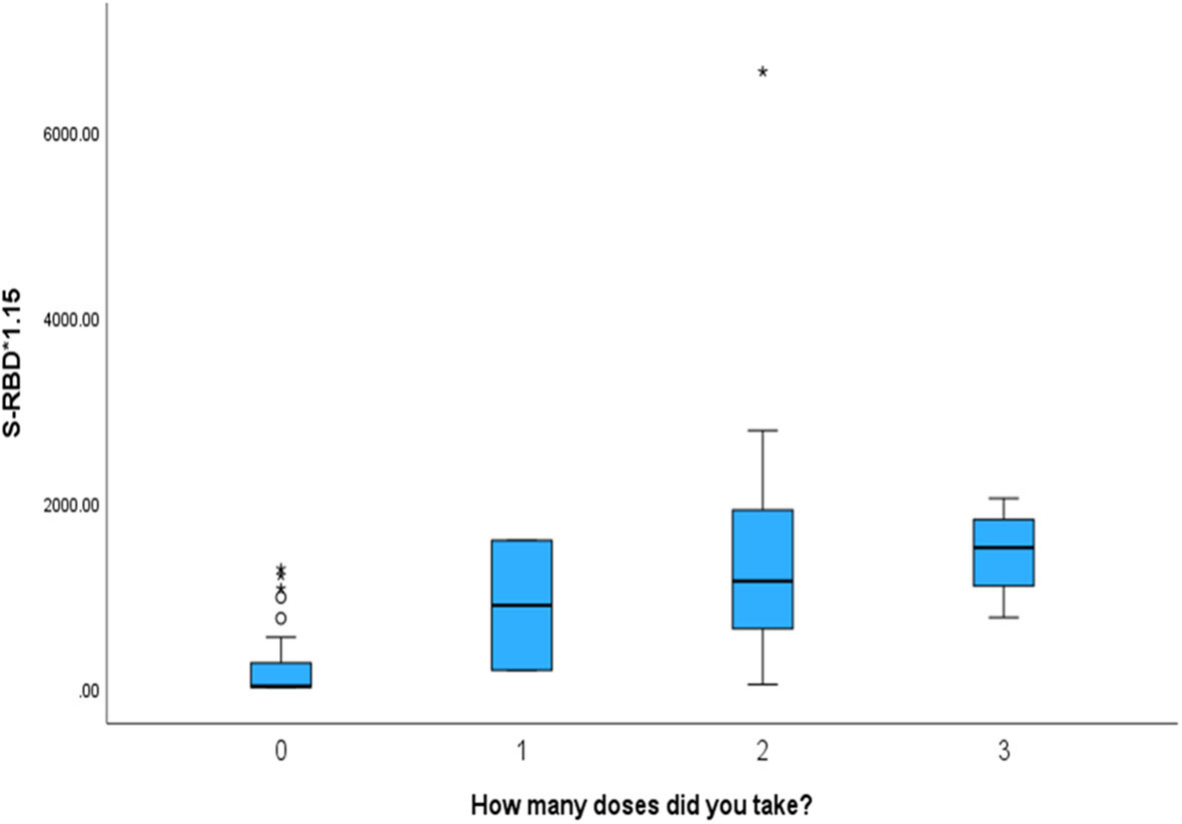
The box plot depicts that mothers who received more vaccination doses against COVID-19 had higher values of S-RBD*1.15 antibodies in the serum of their offspring.

Mothers who received more vaccination doses against COVID-19 had higher values of both antibodies (NTAb*3.31 and S-RBD*1.15) in the serum of their offspring, as shown in the box plots in **Figures 4** and **5**.

Multivariate linear regression analysis was performed using dependent variables NTAb*3.31 and S-RBD*1.15 antibodies and independent predictor variables nationality, infant’s gender, vaccination status against COVID-19, and COVID-19 test status; the multivariate linear regression analysis results indicated that vaccination against COVID-19 remained a potential significant (p < 0.0001) predictor for both NTAb*3.31 and S-RBD*1.15 antibodies after adjusting other predictor variables outlined in **Tables 4** and **5**.

**Table 4:** Multivariate linear regression analysis using the dependent variable NTAb*3.31 levels.

Parameter		Regression Coefficient (β)	P-Value	95% Confidence Interval (CI)	
				Lower Bound	Upper Bound
Nationality	Qatari	140.324	0.592	−379.867	660.514
	Non-Qatari	Reference			
Baby’s gender	Male	9.015	0.969	−458.866	476.896
	Female	Reference			
Have you ever been vaccinated against COVID-19?	Non-vaccinated	−1,026.244	<0.001	−1,506.665	−545.822
	Vaccinated	Reference			
Have you ever tested positive for COVID-19	Negative	223.278	0.362	−262.577	709.134
	Positive	Reference			

R-squared = 0.244 (adjusted R-squared = 0.198); F = 5.33 (p = 0.001).

**Table 5:** Multivariate linear regression analysis using the dependent variable S-RBD*1.15 antibody levels.

Parameter		Regression Coefficient (β)	P-Value	95% Confidence Interval (CI)	
				Lower Bound	Upper Bound
Nationality	Qatari	−82.827	0.742	−582.169	416.516
	Non-Qatari	Reference			
Baby’s gender	Male	−213.409	0.346	−662.539	235.720
	Female	Reference			
Have you ever been vaccinated against COVID-19?	Non-Vaccinated	−1,115.378	<0.001	−1,576.546	−654.211
	Vaccinated	Reference			
Have you ever tested positive for COVID-19?	Negative	43.487	0.853	−422.897	509.870
	Positive	Reference			

R-squared = 0.267 (adjusted R-squared = 0.223); F = 6.02 (p < 0.0001).

## Discussion

COVID-19, caused by SARS-CoV-2, is highly infectious (13). SARS-CoV-2 was first described by Huang et al. (1). Pregnant women and newborns are at-risk groups for COVID-19 (3, 4). Tests for IgG and IgM antibodies for SARS-CoV-2 became available in February 2020. On March 4, 2020, the seventh edition of The New Coronavirus Pneumonia Prevention and Control Protocol for SARS-CoV-2 was released by the National Health Commission of the People’s Republic of China and added serological diagnostic criteria (8).

NTAb*3.31 antibody is a species-specific, monoclonal antibody that targets SARS-CoV-2 and plays a significant role in the prevention of COVID-19 infection as well as virus elimination by inhibiting the interaction of the virus with the host cell receptors, as well as by interacting with phagocytes, natural killer cells, and complement. It is associated with early viral control, highlighting its relevance as a reliable immunological correlate of protection (CoP) against infection. S-RBD*1.15 is a recombinant monoclonal antibody that prevents viral interaction between RBD and angiotensin-converting enzyme 2 (ACE2) receptor, located on the surface of host cells (14). Studies have shown that the mean NTAb*3.31 and S-RBD*1.15 antibody titres of previously infected and vaccinated COVID-19 patients are higher than those of uninfected and vaccinated individuals. Among those infected, patients with moderate-to-severe symptoms had higher mean NTAb*3.31 and S-RBD*1.15 antibody titres than those with mild symptoms. It was also noted that vaccinated individuals had significantly higher NTAb*3.31 and S-RBD*1.15 antibody titres than COVID-19 patients. Further studies are required to investigate the usefulness of NTAb*3.31 and S-RBD*1.15 antibody titres in potential future reinfections with new viral variants (15, 16).

We prospectively investigated the immunological characteristics of newborns delivered to mothers with either previous COVID-19 infection or who received COVID-19 vaccination for the possibility of acquiring transplacental passive immunity in 2021 and 2022. Results from our study revealed significantly higher titres of NTAb*3.31 and S-RBD*1.15 antibodies in newborns’ cord blood whose mothers had previous COVID-19 infection or received COVID-19 vaccination; however, these titres were higher in the case of vaccination than previous infection. This indicates that the rate of transplacental immunity transmission from mothers to their newborns after previous COVID-19 vaccination is higher in those with previous infections. Boelig et al. had similar results, concluding that the rate of transplacental transmission of anti-COVID-19 antibodies is higher in pregnant women with previous COVID-19 vaccination compared to previous COVID-19 infection (17). Our results are similar to those of Lubrano et al., who concluded that vaccinating mothers against COVID-19 is protective for their foetuses/newborns against infection (18, 19).

Another key result from our study is that the more doses of vaccine received, the higher the antibody levels in newborns’ cord blood (p < 0.001). This can be explained by enhanced maternal binding during pregnancy as well as the *in utero* transmission of antibodies from the maternal serum to the foetus. Munoz et al. and Enengl et al. found similar results (20–22). Enengl et al. concluded that more than half of women will be positive for anti-SARS-CoV-2 IgG antibodies at 1–2 weeks after COVID-19 infection. They found that the higher the maternal antibody level, the higher the concentration in the serum of their newborns (22). Consequently, the *in utero* transmission of antibodies has a protective role in neonatal immunity against infection (10, 21–23). Mugo et al. found a positive correlation between maternal and cord blood anti-spike concentration, which suggests that interventions that increase maternal antibody concentrations, such as vaccination, may increase passive immunity and protection against severe COVID-19 in neonates (21). Oz-Alcalay et al. also concluded the same findings of a strong positive correlation between the maternal antibody levels and those in the neonatal serum. Of note, the authors also concluded that the levels of antibodies in the infants’ serum linearly decreased if the mother was vaccinated before 30 weeks of gestation. They recommended that future research studies investigate the effectiveness of booster vaccination doses during pregnancy on the infants’ antibody titres (23). Sturrock et al. found that both maternal infection and vaccination produced an antibody response in 100% of mothers and 92.9% and 93.8% of neonates, respectively. This antibody titre persisted at 6 weeks of age in 95% of neonates. Interestingly, they found that the anti-spike antibody response decreased by almost 25-fold from the first to third trimester of maternal vaccination (24). In continuation of their recommendation, our study and other researchers have shown that the more the maternal vaccination doses, the higher the infants’ antibody titres (17, 24–28).

SARS-CoV-2 vaccination is safe, and published studies have reported no major side effects, especially during the second and third trimesters of pregnancy and during breastfeeding. Conversely, available studies have revealed that infants received specific SARS-CoV-2 antibodies after maternal vaccination (29).

The study has some limitations. Firstly, it is a single-centre study. Secondly, the relatively small sample size limits the statistical power and generalisability of the findings. Additionally, as a prospective observational study, it can only establish associations, not causation. Also, the distribution of participants by vaccination and infection status was not fully detailed, which could introduce bias or complicate the interpretation of subgroup differences. However, the prospective study design, relevance, and the laboratory-based results enhance its strength, power, validity, and generalisability. Studies with large sample sizes are required to reinforce the results of our study and to investigate the mechanism by which the timing of vaccination affects the *in utero* transmission of antibodies (24).

## Conclusions

In our study, we found significantly higher titres of NTAb*3.31 and S-RBD*1.15 antibodies in newborns’ cord blood whose mothers had previous COVID-19 infection or received COVID-19 vaccination; however, these titres were higher in the case of vaccination than previous infection. The more doses of vaccine received, the higher the antibody levels in newborns’ cord blood. This indicates transplacental immunity transmission from mothers to their newborns after previous COVID-19 vaccination or infection.

## Data Availability

The raw data supporting the conclusions of this article will be made available by the corresponding authors, upon reasonable request.
